# Evidence for a dynamic role for mononuclear phagocytes during endometrial repair and remodelling

**DOI:** 10.1038/srep36748

**Published:** 2016-11-09

**Authors:** Fiona L. Cousins, Phoebe M. Kirkwood, Philippa T. K. Saunders, Douglas A. Gibson

**Affiliations:** 1 MRC Centre Inflammation Research, The University of Edinburgh, Queen’s Medical Research Institute, Edinburgh EH16 4TJ.

## Abstract

In women, endometrial breakdown, which is experienced as menstruation, is characterised by high concentrations of inflammatory mediators and immune cells which account for ~40% of the stromal compartment during tissue shedding. These inflammatory cells are known to play a pivotal role in tissue breakdown but their contribution to the rapid scarless repair of endometrium remains poorly understood. In the current study we used a mouse model of menstruation to investigate dynamic changes in mononuclear phagocytes during endometrial repair and remodelling. Menstruation was simulated in *MacGreen* mice to allow visualisation of CSF1R^+^ mononuclear phagocytes. Immunohistochemistry revealed dynamic spatio-temporal changes in numbers and location of CSF1R-EGFP^+^ cells and Ly6G^+^ neutrophils. Flow cytometry confirmed a striking increase in numbers of GFP^+^ cells during repair (24 h): influxed cells were 66% F4/80^+^Gr-1^+^ and 30% F4/80^−^Gr-1^+^. Immunostaining identified distinct populations of putative ‘classical’ monocytes (GFP^+^F4/80^−^), monocyte-derived macrophages (GFP^+^F4/80^+^) and a stable population of putative tissue-resident macrophages (GFP^-^F4/80^+^) localised to areas of breakdown, repair and remodelling respectively. Collectively, these data provide the first compelling evidence to support a role for different populations of monocytes/macrophages in endometrial repair and provide the platform for future studies on the role of these cells in scarless healing.

The human endometrium is a multi-cellular tissue that undergoes repeated cycles of proliferation, differentiation (decidualization), shedding and repair^1^,^2^. Throughout a woman’s reproductive lifetime this cycle of breakdown and repair can occur as many as 400 times and remarkably, this healing usually occurs without development of a ‘scar’. Disturbances in the process of endometrial repair have serious consequences as illustrated by the formation of intrauterine adhesions such as those experienced by patients with Asherman’s syndrome^3^.

In each cycle, in response to progesterone, the endometrium undergoes functional remodelling in order to establish an environment capable of supporting a prospective pregnancy. This terminal differentiation event, decidualization, is associated with vascular remodelling and an increase in the number of endometrial immune cells^4^. In the absence of a conceptus, ovarian progesterone concentrations decrease rapidly and the luminal portion of the endometrium breaks down and is shed during menstruation. Withdrawal of progesterone results in vasoconstriction, focal hypoxia, cytokine release and activation of enzymes that break down the tissue. Examination of human endometrium during menstruation shows it resembles a bloody ‘wound’, with evidence for simultaneous shedding and repair^5^. Resolution of the endometrial ‘wound’ is critical for ongoing reproductive function while dysregulation of menstrual physiology underpins many common gynaecological conditions, such as heavy menstrual bleeding and endometriosis.

Inflammation is a key regulator of wound-healing and studies in experimental models suggest that excess inflammation during healing may promote dysregulated repair and fibrosis/scarring^6^. In contrast, diminished inflammatory responses are associated with rapid, scar-free healing. For example, PU.1 knockout mice, which are depleted in neutrophils and macrophages, have enhanced re-epithelialisation and fibrosis-free healing of cutaneous wounds^7^. Scar-free healing is rare in adult tissues but is observed in foetal life when immune responses to wounds are dampened (reviewed in ref. 8). Interestingly, compared to dermal wounds, oral mucosal wounds have been shown to heal faster and without scarring, which is associated with a reduced inflammatory response^9^. These studies suggest that diminished inflammation is key to promoting scar-free repair which appears at odds with the evidence that menstruation is an inflammatory event^10^,^11^,^12^,^13^,^14^,^15^,^16^,^17^.

Studies using human tissue samples and *in vitro* cultures have suggested neutrophils are the dominant leukocyte during endometrial tissue breakdown^12^,^18^. Studies from the Salamonsen group using a mouse model of ‘menstruation’ reported that neutrophils are increased during breakdown, and that their numbers peak during the repair phase^17^. In the same study, antibody-mediated depletion of Gr-1 positive cells (putative peripheral neutrophils) resulted in delayed repair of the murine endometrium, leading the authors to conclude that neutrophils were essential for endometrial repair^17^. However, these findings are confounded by the fact Gr-1 can be expressed by cells other than neutrophils^19^,^20^.

Macrophages have pleiotropic roles in wound healing including regulation of inflammation, removal of apoptotic tissue and restoration of tissue integrity^21^. Furthermore, the wounds of mice depleted of macrophages exhibit impaired neoangiogenesis and wound closure^22^. Analysis of macrophages in intact cycling mice demonstrated that F4/80 positive cells are abundant in the mouse uterus^23^. CD68^+^ macrophages are abundant in the human endometrium during breakdown *and* repair, and thus may play a key role in tissue clearance and tissue remodelling associated with menstruation. However, the only analysis to date of macrophages in a mouse model of menstruation suggested that F4/80^+^ macrophages are detected distal to the lumen, not associated with areas of tissue remodelling and thus unlikely to be contributing to repair^17^.

In this study we have utilised the *MacGreen* mouse, in which enhanced green fluorescent protein (EGFP) is expressed under the control of the *c-fms* promoter (encodes CSF-1R^24^), to extend our previous studies on endometrial breakdown using a recently refined mouse model of menstruation^25^. Using this model we have extensively characterised different mechanisms responsible for mediating endometrial breakdown and repair processes. We have previously demonstrated that hypoxia^26^, apoptosis and expression of matrix metalloproteinases (MMP3, MMP9) are temporally and spatially regulated during endometrial breakdown^27^ and that re-epithelialisation is mediated by epithelial proliferation and mesenchymal to epithelial transition during endometrial repair^25^. These studies highlight the complex and tightly-regulated remodelling that occurs within the uterus during post-menstrual repair. In the current study, we have assessed changes in intrauterine populations of cells of the mononuclear phagocyte system to elucidate the role of these cells in endometrial repair and remodelling. We found that ‘menstruation’ was associated with a transient influx of classical monocytes and monocyte-derived macrophages which associated with regions of tissue breakdown and repair. This study provides the first evidence that monocytes/macrophages have a predominant role in endometrial remodelling following menstruation. These data provide new insights into macrophage dynamics in the uterus and provide a novel paradigm for investigating the role of macrophages in promoting scarless healing.

## Results

### Endometrial breakdown and repair is associated with dynamic spatio-temporal changes in immune cell populations

**Figure 1:**
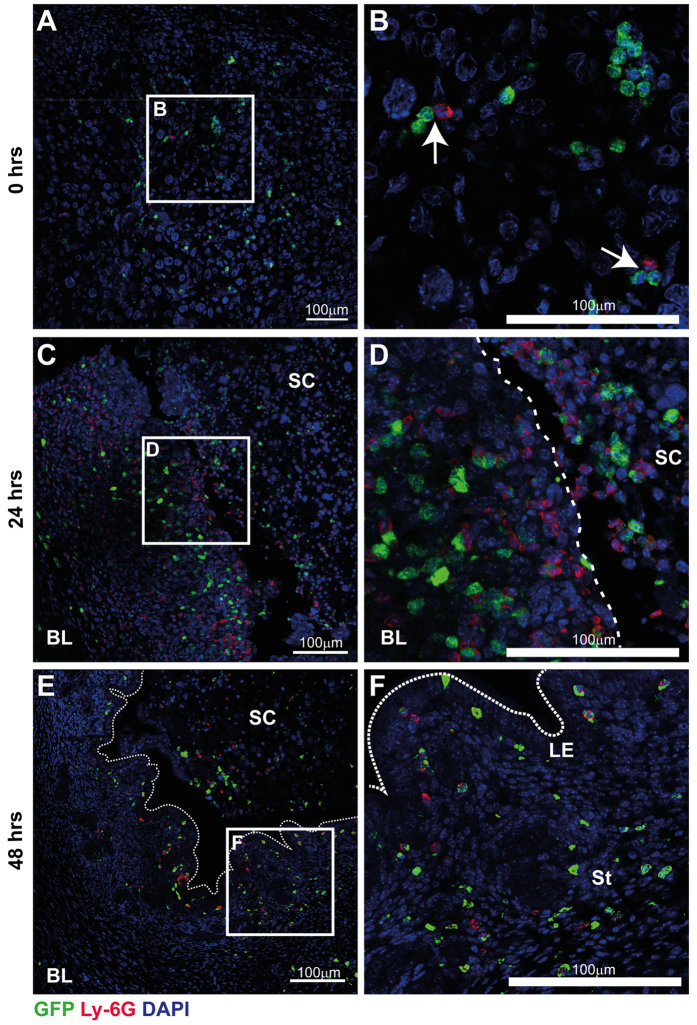
Immune cell dynamics during endometrial breakdown, repair and remodelling. The population of GFP^+^ immune cells during menstruation was assessed by immunohistochemistry in uterine tissue recovered prior to, and 24- and 48-hours after withdrawal of progesterone. (**A**) Prior to endometrial breakdown (time of progesterone withdrawal; 0 hrs), in a fully decidualized mouse uterine horn, macrophages (GFP; green) and neutrophils (Ly6G; red) were detected diffusely scattered throughout the tissue. (**B**) Ly6G^+^ neutrophils were scarce but were detected in close proximity to macrophages (white arrows). (**C**) During endometrial breakdown and repair (24 hrs after withdrawal of progesterone), a dramatic influx of immune cells is apparent throughout the uterus. Discrete but closely adjacent populations of macrophages (GFP^+^) and neutrophils (Ly6G^+^, red) were identified in both basal layer (BL) and shed cells (SC). Notably both cell types were observed in close proximity to the repairing luminal surface (dashed line). (**D)** Macrophages and neutrophils were commonly detected clustered in areas of tissue detachment and breakdown. (**E)** At 48 hrs, endometrial repair is complete and re-epithelisation of the luminal surface was apparent (dotted line). Neutrophil numbers appeared to decrease during tissue remodelling and at this time, few Ly6G^+^ neutrophils were detected. (**F**) GFP^+^ macrophages were still detected within the uterus and were localised to areas surrounding the newly repaired luminal epithelium (dotted line; LE), the stroma (St) and within the residual shed cell mass (SC). Nuclear counterstain DAPI (blue), scale bars 100 μm.

In *MacGreen* mice, cells of the mononuclear phagocyte system express EGFP upon activation of the *c-fms* (also known as *Csf1r*) promoter. However, although the original publication reported expression of F4/80^+^ macrophages in diverse tissues including liver, lung, bone marrow and trophoblast^24^, subsequent investigations showed that EGFP was expressed outside the mononuclear phagocyte system by granulocytes identified by a Gr-1 antibody^20^. Thus, we first used immunohistochemistry to investigate the nature of GFP^+^ cells in the mouse uterus during tissue breakdown and repair. As described previously^25^, menstruation was induced by withdrawal of progesterone and tissues were assessed at various time-points thereafter. Initial experiments, focussing on 4 hours post progesterone withdrawal, identified GFP^+^ cells in mouse uterine tissues. Importantly, these GFP^+^ cells lacked expression of the neutrophil-specific Ly6G antigen, which can also be detected by the Gr-1 antibody, ruling out the possibility that the GFP^+^ cells represented neutrophils (Supplementary Figure 1). Similarly, they did not stain with *Dolichos biflorus* agglutinin (DBA) lectin, suggesting they did not represent natural killer (NK) cells (Supplementary Figure 1). Notably, very few GFP^+^ cells were detected in mouse uterine tissues from females experiencing normal estrus cycles even though expression of F4/80 was readily detected (Supplementary Figure 2). These data were extended by exploring the expression of GFP and Ly6G at three key time points; 0 hours (prior to menses), 24 hours (breakdown and repair) and 48 hours (remodelling) after withdrawal of progesterone. At 0 hours, GFP^+^ cells were detected throughout the decidualized tissue (Fig. 1A). Few Ly6G^+^ neutrophils were detected but they appeared in close proximity to GFP^+^ cells (Fig. 1B). By 24 hours, a striking influx of GFP^+^ cells and Ly6G^+^ cells that did not co-localise was detected in areas of tissue undergoing active breakdown and repair (Fig. 1C). Elicited leukocytes accumulated close to the denuded luminal surface (Fig. 1C; dashed line) and within shed endometrial tissue (Fig. 1D). By 48 hours, the endometrial repair appeared complete, characterised by a fully re-epithelialized luminal surface. In parallel, very few neutrophils were detected, suggesting that the acute phase of tissue inflammation appeared to have abated (Fig. 1E). Notably, GFP^+^ cells were still present consistent with an active role for myeloid cells during tissue remodelling occurring at this time point.

### A significant influx of GFP^+^ cells occurs 24 hours after withdrawal of progesterone

To further assess the phenotype of the GFP^+^ cell populations during menses, uterine tissues were digestion and isolated cells were analysed by flow cytometry. GFP^+^ cells were most abundant 24 hours after withdrawal of progesterone (Supplementary Figure 3) and this time point was used for further analysis.

**Figure 2:**
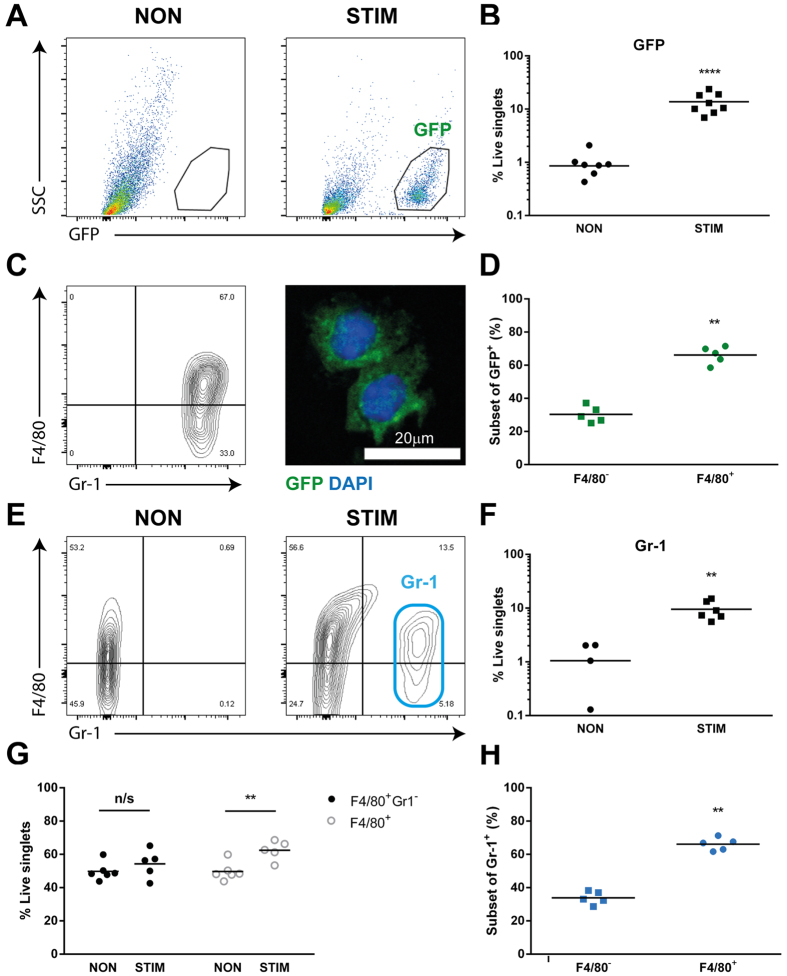
Immunophenotyping of uterine macrophages 24 hours after withdrawal of progesterone. GFP^+^ cell populations present in the uterus were characterised by flow cytometry of tissue digested from both control (non-stimulated/non-decidualized) and decidualized horns. (**A**) In *MacGreen* mice, few GFP^+^ cells were detected in non-stimulated uterine horns (non), which was in marked contrast to the abundant GFP^+^ cells in the decidualized horn (stim). (**B**) Analysis of proportion of influxing cells revealed a significant increase in GFP^+^ cell populations (p < 0.001) in stimulated uterine horns (n = 7). GFP^+^ cells (see **A**) were gated for further analysis and expression of F4/80 and Gr-1 was assessed. (**C**) The majority of GFP^+^ cells were F4/80^+^ and almost all expressed Gr-1. Isolated GFP^+^ cells had mononuclear morphology as determined by immunofluorescence (green; GFP, blue; DAPI). (**D**) F4/80 expression within the GFP^+^ cell population was detected in the majority of cells (n = 5, p < 0.01). (**E**) F4/80 and Gr-1 expression in non- and stimulated horns in wildtype (WT) mice was assessed. (**F**) In WT mice Gr-1^+^ cells were significantly increased in stimulated compared to control horns (n = 4–6, p < 0.01). (**G**) F4/80^+^ Gr-1^−^ populations were unchanged in non and stimulated uterine horns but the proportion of total F4/80^+^ cells was increased in stimulated compared to control horns (n = 5–6, p < 0.01). (**H**) The majority of influxing Gr-1^+^ cells were F4/80^+^ (n = 5, p < 0.01).

Consistent with immunohistochemical analysis of non-decidualized uterine horns, flow cytometric analysis detected very few GFP^+^ cells present in non-decidualized uterine horns from *MacGreen* mice (Fig. 2A; Supplementary Figure 2). In contrast, GFP^+^ cells were abundant in decidualized tissue undergoing active endometrial repair (Fig. 2A). Quantitation of cells confirmed the proportion of GFP^+^ cells was significantly increased in stimulated (~14%) compared to control (<1%) uterine horns (Fig. 2B; n = 7, p < 0.001). Further analysis revealed that GFP^+^ cells uniformly expressed Gr-1, while expression of F4/80 was more heterogeneous (Fig. 2C and Supplementary Figure 2). Thus the major phenotype of the influxing GFP^+^ cells was F4/80^+^Gr-1^+^ (66%; Fig. 2D; n = 5, p < 0.01) while the remainder were F4/80^−^Gr-1^+^ (30%; Fig. 2D). Immunofluorescence of isolated GFP^+^ cells revealed mononuclear morphology consistent with low SSC/granularity detected by flow cytometry (Fig. 2A,C). Given that the Gr-1 antibody we used detects both the Ly6C and Ly6G antigens and no GFP^+^ cell expressed Ly6G (Fig. 1), the high expression of Gr-1 detected by flow cytometry suggested GFP^+^ cells shared characteristics of ‘classical’ (inflammatory) Ly6C^+^ monocytes/macrophages.

Given the striking expression of Gr-1 in almost all influxing GFP^+^ cells, uterine immune cell populations in wild type (WT) mice were also assessed by flow cytometry (Fig. 2E–H) to independently verify the cell phenotype observed in *MacGreen* mice. Consistent with results obtained with *MacGreen* mice, a striking influx of Gr-1^+^ cells was detected in stimulated uterine horns that was absent in non-stimulated horns (Fig. 2E,F), accounting for around 10% of live cells in WT mice (Fig. 2F; n = 4–6, p < 0.01). Notably, when all live singlet cells were analysed, as opposed to examining only GFP-expressing cells, two F4/80^+^ populations were apparent; F4/80^+^Gr-1^−^ and F4/80^+^Gr-1^+^. F4/80^+^ Gr-1^−^ cells were present in both non- and stimulated uterine horns but appeared unchanged by treatment (Fig. 2G), whereas F4/80^+^Gr-1^+^ cells were only detected in stimulated uterine horns. There was a significant increase in frequency of total F4/80^+^ cells in stimulated vs non-stimulated horns (Fig. 2G; n = 5, p < 0.01) which represented the accumulation of Gr-1^+^ cells. This was also confirmed by live cell singlet gating of F4/80^+^ cells in *MacGreen* mice (Supplementary Figure 4B). Indeed, as in *MacGreen* mice, the majority of Gr-1^+^ cells in the WT uterus (66%) were F4/80^+^ (Fig. 2H; n = 5, p < 0.01). Taken together, these data suggest that the majority of GFP^+^ cells recruited to the uterus during endometrial repair and remodelling are cells of the monocyte/macrophage lineage.

### Distinct populations of monocytes/macrophages associate with areas of endometrial repair and remodelling

**Figure 3:**
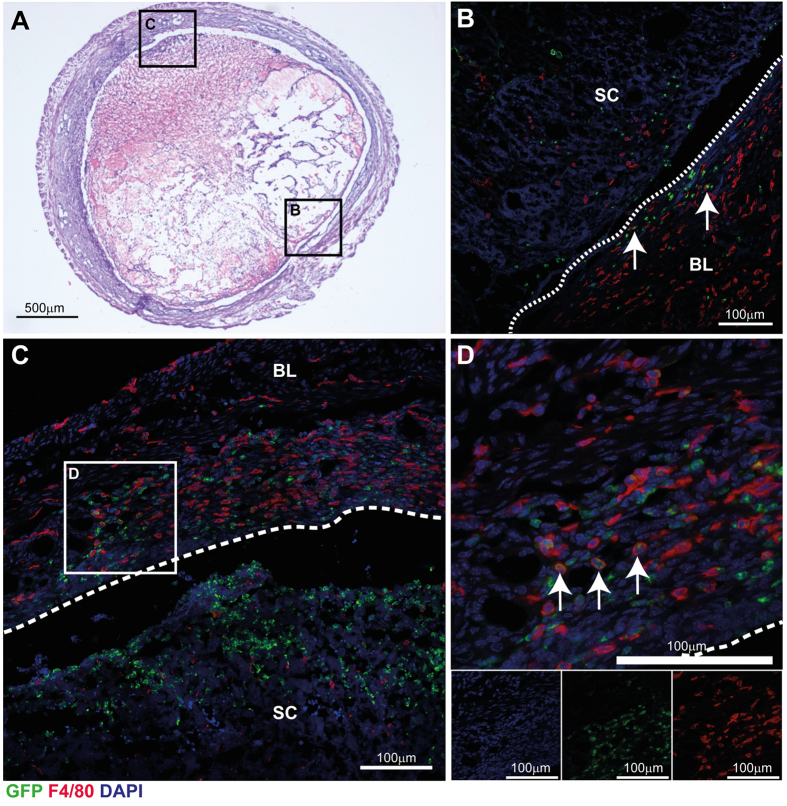
Distinct populations of macrophages associate with areas of tissue at different stages of the repair process. At 24 h after withdrawal of P, populations of both GFP^+^ macrophages and immunopositive for the mouse macrophage surface marker F4/80 (red) were abundant within the tissue. (**A**) Gross morphology of uterine horn 24 hours after withdrawal of progesterone, cross-section of uterine tissue stained with H&E; at this time concomitant tissue breakdown of shed cell mass and repair of basal endometrium is apparent. (**B**) In areas of re-epithelialized (repaired; dotted line) endometrium, populations of F4/80^+^ macrophages are abundant in the basal layer (BL) of the endometrium but scarce within the shed cells (SC) and tissue. GFP positive cells (green) were largely detected exclusive of F4/80 staining with few cells expressing both markers (white arrows). (**C**) In areas of tissue undergoing repair, F4/80^+^ macrophages were observed throughout the stromal compartment in both the basal layer (BL) and close to the denuded epithelial surface (dashed line, 3C and 3D) but not in shed cells (SC). GFP^+^ cells were abundant in shed tissue and did not co-localise with F4/80 staining. (**D**) In contrast, double positive cells were abundant in areas where the luminal surface was undergoing repair or was exposed to the lumen (3C and 3D; white arrows). Nuclear counterstain DAPI (blue), scale bars 100 μm.

Given the apparent heterogeneity of macrophage populations identified by flow cytometry, detailed immunohistochemistry analysis was performed on tissue sections of uterine horns from *MacGreen* mice 24 hours after withdrawal of progesterone. At this time point concurrent tissue breakdown, repair and remodelling are apparent (Fig. 3A). Consistent with flow cytometry results, immunohistochemistry revealed striking evidence for three putative populations of immune cells present during endometrial repair; GFP^+^F4/80^−^, GFP^-^F4/80^+^ and GFP^+^F4/80^+^ cells, which associated with spatially discrete regions of breakdown, repair and remodelling (Fig. 3). In regions of repaired endometrium, epithelial integrity was restored and the re-epithelialized luminal surface is apparent (Fig. 3B; dotted line). In the endometrial stroma underlying the luminal epithelium, F4/80^+^ macrophages were abundant throughout the basal layer (BL), however most of these lacked GFP expression and only very few double positive cells were detected (white arrows). F4/80^+^ and GFP^+^ cells were present throughout the shed tissue but staining did not appear to co-localise.

Clustering of GFP^+^F4/80^−^ cells was detected in areas of decidual detachment and tissue breakdown, while GFP^-^F4/80^+^ cells were detected predominantly in the basal layer underlying repairing areas of tissue and exhibited an elongated morphology (Fig. 3C; BL). GFP^+^F4/80^+^ cells were detected localised to areas of active repair and remodelling next to denuded epithelial surface (Fig. 3D; arrows). Taken together, the temporal and spatial association of the three phenotypically identifiable populations of monocytes/macrophages is entirely consistent with the highly plastic nature of these cells and the ability of the local environment to imprint their phenotypic and functional characteristics.

### Temporal analysis of ‘classical’ monocytes/macrophages during endometrial repair

**Figure 4:**
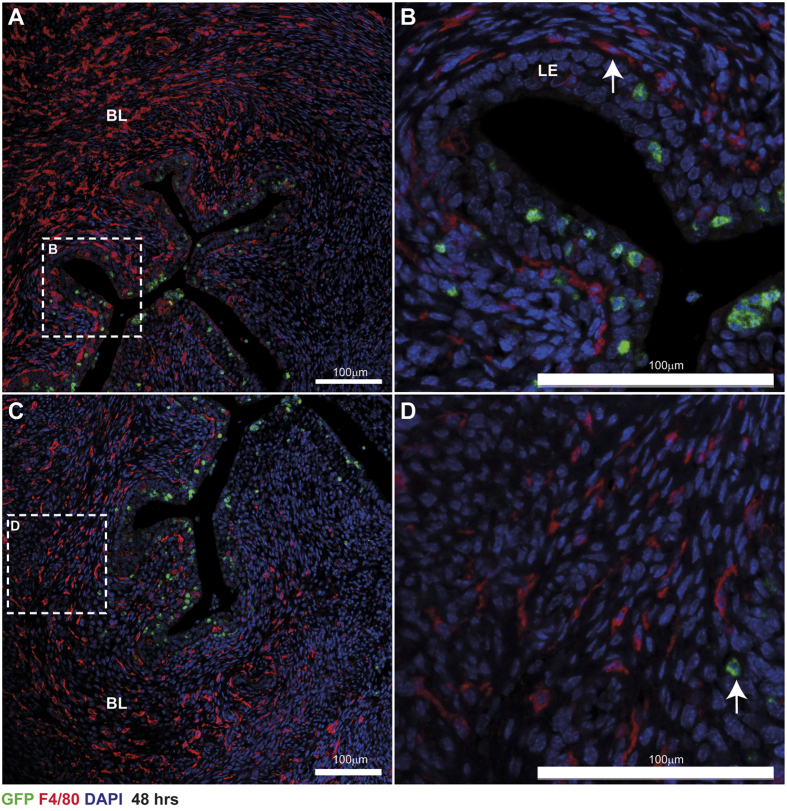
The phenotype of mononuclear phagocytes following endometrial repair. (**A**) Forty-eight hours after withdrawal of P, epithelial integrity had been restored although remodelling of the tissue was incomplete. GFP^+^ macrophages (green) were located in the stroma proximal to the repaired/repairing luminal epithelium and embedded within the luminal epithelium (LE, High power inset; (**B**)). Abundant F4/80 positive macrophages were identified throughout the basal layer (BL) of the stromal compartment and in the remodelling functional layer (**A**,**C**) but they were not GFP^+^. In the basal layer of the tissue F4/80^+^ cells are abundant and have a mature morphology (**D**) few GFP^+^ cells were detectable within this part of the tissue (arrow). BL; basal layer, LE; luminal epithelium, SC; shed cells. Nuclear counterstain DAPI (blue), scale bars 100 μm.

Expression of GFP and F4/80 was also assessed in uterine tissue from *MacGreen* mice 48 hours following withdrawal of progesterone by immunofluorescence. Assessment of macrophages by GFP and F4/80 staining revealed a striking pattern of expression characterised by abundant F4/80^+^ cells throughout the endometrial stroma and clustering of GFP^+^ cells within the stroma proximal to the repaired luminal epithelium and embedded within the luminal epithelium (Fig. 4). In contrast to the 24 hour time point, staining for F4/80 and GFP did not appear to overlap and no double-stained cells were detected (Fig. 4B). The majority of cells detected were mature F4/80^+^ cells located in basal layer of the tissue (Fig. 4C), few GFP^+^ cells were detected in this area of the tissue (Fig. 4D; arrow). This staining pattern was strikingly similar to 0 hour tissue in which the majority of cells were immunopositive for F4/80 but few GFP^+^ cells were detected in the tissue (Supplementary Figure 5).

**Figure 5:**
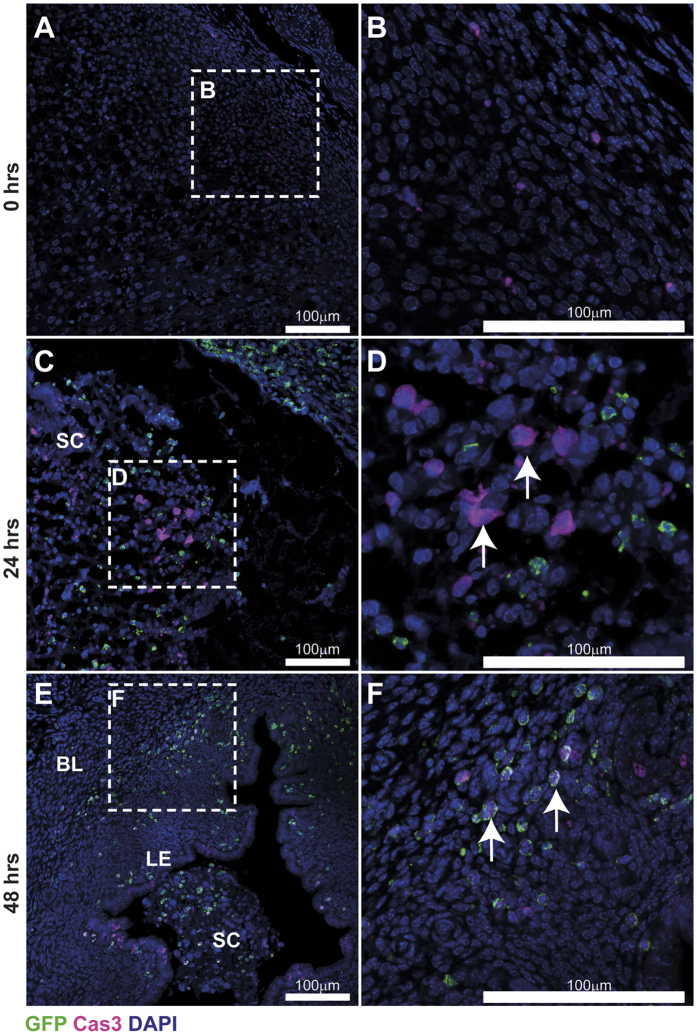
Apoptosis within uterine tissue across a time course of endometrial repair. The expression of cleaved caspase 3 was assessed by immunohistochemistry in stimulated uterine horns recovered 0, 24 and 48 hours after withdrawal of progesterone. Very few cleaved caspase-3-positive cells (violet) could be detected prior to progesterone withdrawal (**A**,**B**). At 24 hours, during endometrial breakdown and repair, cleaved caspase-3-positive apoptotic cells (arrows) were most abundant in the shed cell mass (SC) and were detected in association, but not co-localised with GFP^+^ cells (green; **C**,**D**). By 48 hours, cleaved caspase-3-positive apoptotic cells were present in the luminal epithelium, stroma underlying the repaired epithelium and the residual shed cell (SC) mass. Double positive staining for GFP (green) and Caspase 3 (violet) was detected throughout the tissue (**E**,**F**) in cells within the stroma (white arrows) and the luminal epithelium. BL; basal layer, LE; luminal epithelium, SC; shed cells. Nuclear counterstain DAPI (blue). BL; basal layer, LE; luminal epithelium, SC; shed cells. Nuclear counterstain DAPI (blue), scale bars 100 μm.

We next assessed the timeframe for macrophage apoptosis across endometrial breakdown and repair (Fig. 5) by staining for cleaved caspase 3 in uterine tissues recovered 0, 24 and 48 hours after withdrawal of progesterone. Few caspase 3-positive cells were detected at 0 hours (Fig. 5A,B). At the 24 hour time-point the majority of Caspase 3-positive cells were detected in decidual cells in the shed tissue (Fig. 5C) but no co-localisation of GFP and Caspase-3 was detected (Fig. 5D arrows). At the 48 hour time-point apoptotic cells were detected in the luminal epithelium (LE), in the stroma underlying the repaired epithelium and within the residual shed tissue in the luminal cavity (Fig. 5E; SC). Notably, co-localisation of caspase 3 within all GFP^+^ cells was detected suggesting they were apoptotic and/or involved in the clearance of apoptotic debris. Caspase 3 and GFP positive cells were detected both in the residual shed cells (Fig. 5E; SC) and within the functional endometrium (Fig. 5F; arrows). These data are consistent with programmed cell death of ‘classical’ monocytes following completion of endometrial repair.

## Discussion

The endometrium undergoes rapid, scar-free healing in each menstrual cycle. Endometrial breakdown and repair is associated with overt inflammation and an influx of inflammatory cells including neutrophils and macrophages. Studies in human tissues have detected a progressive increase in numbers of macrophages during the secretory phase of the cycle that peaks during menstruation, during which time they may represent up to 15% of the leukocyte population^28^. Menstruation occurs naturally in only a few species, such as higher primates, and thus *in vivo* studies into the regulation of endometrial repair are limited. We recently updated a modified murine model of artificially-induced menses to study endometrial repair mechanisms. Our analysis of this model shows that it recapitulates key features of the human cycle including transient hypoxia, cytokine release, activation of matrix metalloproteinases and infiltration of leukocytes^25^,^26^,^27^. In the current study, we have modelled menstruation in *MacGreen* mice and performed detailed analysis with confocal microscopy and flow cytometry to elucidate dynamic changes in populations of cells of the mononuclear phagocyte system during endometrial breakdown, repair and remodelling.

The differentiation of macrophages from myeloid progenitors is co-ordinated by macrophage colony-stimulating factor (CSF-1)^29^ which binds to cells that express the CSF-1 receptor (CSF-1R) encoded by the *c-fms* proto-oncogene. Sasmono *et al*. have incorporated the enhanced green fluorescent protein (EGFP) reporter gene into the proximal promoter of the *c-fms* gene^24^ to visualise macrophages in all body tissues. In the current study, combined analysis of F4/80- and GFP-expressing cells in the *MacGreen* mouse uterus identified three putative populations present during endometrial repair; GFP^+^F4/80^−^, GFP^+^F4/80^+^ and GFP^-^F4/80^+^. Flow cytometry analysis of GFP^+^ cells revealed that these cells were also positive for Gr-1. Gr-1^+^ cells had low side-scatter and morphological analysis by immunofluorescence confirmed that isolated GFP^+^ cells were mononuclear cells (Fig. 2 and Supplementary Figure 4). Expression of F4/80 was detected in the majority of GFP^+/^Gr-1^+^ cells by flow cytometry, consistent with differentiation of monocytes into macrophages within endometrial tissue. On this basis, the myeloid cells identified during endometrial repair were characterised as influxing ‘classical’ (inflammatory) monocytes (GFP^+^F4/80^−^Gr-1^+^) and monocyte-derived macrophages (GFP^+^F4/80^+^Gr-1^+^). In addition, a population of F4/80^+^ cells was identified that did not express either GFP or Gr-1, and given the abundance of these did not change during endometrial repair, these could represent putative tissue-resident macrophages. Thus, previous studies using single markers to assess macrophages (F4/80, *MacGreen*) or neutrophils (Gr-1) in the uterus now require reinterpretation. These studies have reported that neutrophils are critical to endometrial repair based on depletion using the RB6-8C5 anti-Gr-1 antibody^17^,^30^. However, as RB6-8C5 has specificity for Ly6C, this would undoubtedly target classical blood monocytes and their descendants in tissue^31^,^32^. Indeed, we found uterine GFP^+^ monocyte/macrophages to be Gr-1^+^ but Ly6G^−^ using the same RB6-8C5 clone. Thus, our data show that depletion using this antibody may result in a wider depletion of mononuclear phagocytes including ‘classical’ monocytes and macrophages (as well as neutrophils) consistent with the established importance of mononuclear phagocytes in wound healing.

Monocyte recruitment occurs in response to ‘sterile’ inflammation during endometrial repair, similar to inflammatory responses to injury in the cardiovascular system. In a mouse model of myocardial infarction, Ly6C^high^ monocytes are initially reported to facilitate removal of dead tissue followed by a second wave of Ly6C^low^ monocytes that promote resolution of inflammation and tissue repair^33^. In the current study, we found spatio-temporal regulation of ‘classical’ monocytes during endometrial repair. However, in contrast to the linear, biphasic progression of inflammation reported in myocardial infarction, *simultaneous* breakdown and repair is apparent during menstruation. Thus we found abundant ‘classical’ monocytes in areas of tissue breakdown, as well as concomitant monocyte-derived macrophages in areas of repairing endometrium. In addition, putative tissue-resident macrophages appeared to be spatially restricted; GFP^-^F4/80^+^ cells were detected only in association with areas of repaired, re-epithelialized endometrium. We and others have previously speculated that factors secreted from the shed tissue may promote repair^5^,^25^,^34^. In a detailed analysis of human endometrial tissue, Gaide Chevronnay *et al*. used laser capture microdissection to assess the transcriptional profile of shed and basal tissue during menstrual breakdown^34^. They found that shed tissue had a distinct transcriptional profile characterised by genes essential for the regulation of apoptosis, extracellular matrix remodelling and factors that could regulate immune cell function^34^. Furthermore, we have previously shown that the luminal epithelium and sub-luminal stroma is exposed to hypoxia during endometrial breakdown and that time-dependent changes in tissue hypoxia correlate with the regulation of angiogenesis-associated genes (*Vegfa*) as well as genes that regulate leukocyte chemotaxis (*Cxcl12*)^26^ which may impact on recruitment and function of monocyte/macrophage populations during endometrial repair. These data suggest that distinct microenvironment signals from both the shed decidual tissue and the repairing endometrium may program the function and differentiation of monocytes during endometrial repair.

In many tissues in *MacGreen* mice, the expression of EGFP corresponds to F4/80^+^ cell populations. However, we found that the majority of F4/80^+^ cells detected in the uterus did not express GFP, particularly in uteri from intact cycling mice, unstimulated uterine horns (Supplementary Figures 2 and 4) or following completion of endometrial repair (Fig. 4A). Interestingly, the proportion of GFP^−^F4/80^+^ cells did not appear to change between unstimulated and stimulated uteri during endometrial repair (Supplementary Figures 2 and 4). Although the limited co-expression of GFP and F4/80 we detected in the uterus was surprising, it is consistent with previous reports that macrophages are not solely dependent on CSF-1. For example, there is incomplete depletion of macrophages in mice lacking functional CSF-1 (*op/op* mice) or in *Csf1r* knockout mice^35^,^36^. Furthermore, our analysis of uterine tissues from intact cycling mice revealed an abundance of F4/80^+^ cells with limited expression of GFP (Supplementary Figure 2) which suggests that uterine tissue-resident macrophages do not normally express CSF-1R. Removal/reduction of CSF-1 promotes quiescence of bone marrow derived macrophages *in vitro*^37^. Given the homeostatic nature of the tissue resident macrophages we have detected during endometrial repair, we speculate that these cells are quiescent and persist independent of the actions of CSF-1. In intact cycling mice, macrophages (identified by F4/80) are abundant in the uterus but macrophage numbers are reported to be reduced following antibody depletion of CSF-1 or in *op/op* mice, indicating CSF-1 plays a major role in macrophage recruitment and differentiation in the uterus^23^. In contrast, antibody depletion of CSF-1R in *MacGreen* mice reported by MacDonald *et al*., demonstrated loss of macrophages (detected by GFP expression) from several tissues but *not* the uterus^38^. These studies offer seemingly contradictory conclusions and suggest that uterine macrophages are both CSF-1-dependent and CSF-1R-independent. This may indicate that uterine macrophages require CSF-1 but acting either via an alternative receptor or through indirect regulation via cross-talk from other cells within the endometrium. A key caveat to the interpretation of these studies is that they were performed in intact cycling mice. In contrast, the current study examined the uterus in response to the physiological stress of menses. Our results provide novel data that demonstrate both an influx of GFP^+^ cells and the presence of F4/80^+^ macrophages that do not express GFP (presumptively CSF-1 independent) are present during endometrial repair. Taken together these data suggest that more than one mechanism may be responsible for macrophage maintenance in the uterus and this population arises and may be maintained by overlapping, redundant mechanisms depending on the state of the tissue.

We did not detect any difference in intensity of GFP/Gr-1 amongst influxing cells, although it is possible that an equivalent Gr-1^low^/Ly6C^low^ ‘non-classical’ monocyte population contributes to the population of tissue resident macrophages associated with the repaired endometrium. Indeed, CX_3_CR1^+^ monocytes are reported to be Gr-1^−^ 39 and therefore could account for some of the GFP^-^F4/80^+^ cells we detected in the uterus. One possible growth factor that could contribute to maintenance of GFP^-^F4/80^+^ cells is GM-CSF (CSF-2). In development, alveolar macrophages develop from foetal monocytes and differentiate into long-lived tissue resident macrophages via a GM-CSF-dependent mechanism^40^. GM-CSF expression is reported to be localised to endometrial epithelial cells in the mouse uterus^41^. Interestingly, GFP^-^F4/80^+^ cells were only detected in areas of tissue with restored epithelial integrity which may suggest that epithelial cell-derived GM-CSF could promote differentiation of this subset of macrophages. The apparent divergence of CSF-1R-dependence amongst macrophages in the endometrium may provide a novel strategy for targeting endometrial macrophage subsets which may be a beneficial therapeutic target in the treatment of menstrual disorders.

**Figure 6:**
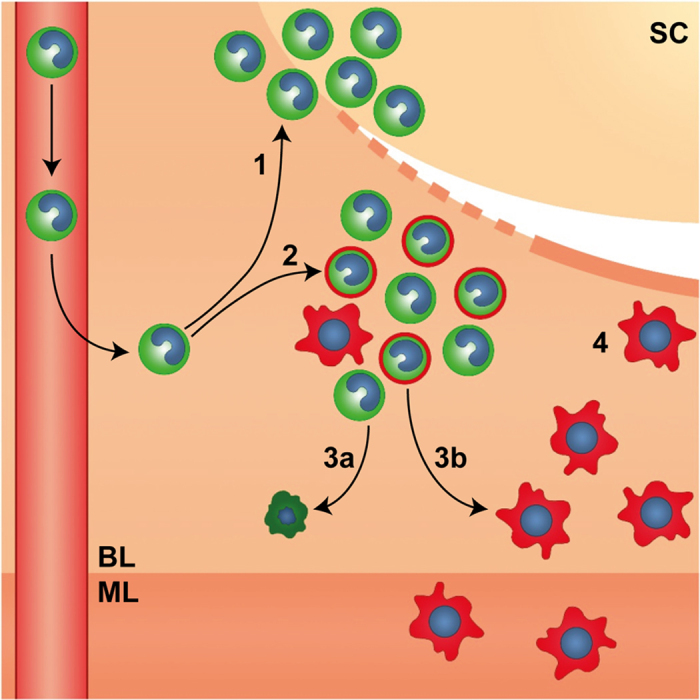
The role of mononuclear phagocytes during endometrial repair. Endometrial repair is rapid and occurs concurrently with tissue breakdown and clearance of shed cells (SC). Endometrial repair is associated with a dynamic influx of ‘classical’ monocytes from the circulation in response to the ‘wounding’ stimulus of menses which differentiate in response to spatially distinct signals within the tissue. 1. GFP^+^ monocytes (green) cluster in areas of tissue breakdown and shedding. 2. A mixed population of GFP^+^ ‘classical’ monocytes and GFP^+^F4/80^+^ monocyte-derived macrophages (green/red) cluster in areas of active repair and remodelling in close proximity to denuded stromal surfaces (dotted line). 3a. GFP^+^ monocytes may undergo apoptosis and be cleared from the tissue following resolution of inflammation or 3b. undergo differentiation into tissue resident macrophages. 4. F4/80^+^ tissue resident macrophages (red) are associated with newly re-epithelialized areas of repaired tissue (solid line) and are detected within the basal (BL) and myometrial layer (ML) of the uterus.

GFP^+^F4/80^+^ cells were only detected transiently in the uterus. To assess the fate of GFP^+^ cells we investigated cellular apoptosis by Caspase 3 staining and found that apoptotic GFP^+^ cells were only detected 48 hours after progesterone withdrawal. Thus, GFP^+^ cells did not undergo apoptosis until endometrial repair was complete providing further evidence that signals generated by the remodelling tissue may contribute to the maintenance/differentiation of influxing monocytes. Based on our new findings, we speculate that following influx into the endometrium, the fate of GFP^+^ monocytes follows one of two pathways: either differentiation into monocyte-derived macrophages or programmed cell death following resolution of inflammation (summarised in Fig. 6). Further lineage tracing studies are required to determine if GFP^+^ cells contribute to tissue-resident macrophages (GFP^-^F4/80^+^) in the endometrium but the characterisation described in this study suggests these cells are abundant and persist independent of CSF-1 following endometrial repair.

The findings of the current study highlight the limitation of ‘definitive’ markers for immune cell subpopulations and highlight the importance of complementing immuno-phenotyping by flow cytometry with immunohistochemistry analysis to appreciate *in vivo* tissue dynamics with particular regard to cellular location and association with functional processes. Using a mouse model of menstruation and the *MacGreen* mouse we have uncovered novel roles for the mononuclear phagocyte system in the regulation of endometrial function. We confirm previous reports describing F4/80^+^ cells in the uterus but provide new data that suggest this population is quiescent and characteristic of a tissue-resident, mature macrophage population. Importantly, we have identified a transient population of influxing CSF-1R/GFP^+^ monocytes that are present as ‘classical’ monocytes or monocyte-derived macrophages which associate with spatio-temporal regulation of endometrial repair and remodelling. In-depth characterisation and phenotyping of these uterine macrophage populations could lead to new insights into the role of macrophage heterogeneity in the regulation of tissue repair and scarless healing.

## Methods

### Animals

Generation of the *MacGreen* mouse has been described previously^24^. Founder stocks of the *MacGreen* mouse were obtained from Dr. Bernadette Dutia and Professor David Hume (Roslin Institute, University of Edinburgh). Animals were genotyped at weaning as described previously^42^.

### Mouse model of menstruation and repair

All animal procedures were carried out in accordance with the Animal Welfare and Ethical Review Body (AWERB) and under licensed approval from the UK Home Office. A mouse model of menstruation and repair that mimicked key features of menstruation in women was previously validated^25^,^26^. Briefly, *MacGreen* mice between 8–10 weeks of age were ovariectomised on day 0. Mice received daily injections of β-estradiol (E2) in sesame seed oil (100 ng/100 μl, days 7–9). A progesterone (P)-secreting pellet was placed sub-cutaneously on day 13; mice also received daily injections of sub-cutaneous injections of E2 (5 ng/100 μl, days 13–15). On day 15, decidualization was induced by stimulation of one horn using sesame seed oil (20 μl) dispersed into the uterine lumen (‘stimulated’). The contra-lateral horn acted as a control ‘non-stimulated’ horn. P withdrawal was induced 90 hours later. Uteri were collected at time of P withdrawal (0 hours) or 8, 24 and 48 hours thereafter. Uteri were fixed in either 10% neutral buffer formalin (NBF) or 4% paraformaldehyde (PFA) or transferred to PBS prior to tissue digest.

### Immunohistochemistry

Haematoxylin and Eosin (H&E) staining was performed according to standard methods. Double immunofluorescence was carried out with antibodies directed against GFP, Ly6G (neutrophil marker), DBA-lectin (uNK cell marker), F4/80 (macrophage marker) or cleaved caspase-3 (apoptosis marker). Details of antibodies are provided in supplementary table 1. Primary antibodies were incubated at 4 °C overnight. Antigen detection was performed using Tyramide signal amplification (Perkin Elmer) system followed by nuclear counterstaining with DAPI (4′,6-Diamidino-2-phenyl-indole dihydrochloride). Serial, transverse 5 μm sections were cut and a minimum of 4 sections of each uterine horn per mouse were examined by immunhistochemistry. Images are representative staining of sections examined from n = 4–6 mice per time point (T0, n = 4 mice; T24, n = 6 mice; T48, n = 4 mice). Images were captured using a LSM 710 Confocal microscope (Zeiss) at x40 magnification. Zen 2011 software was utilised to create tiled images.

### Flow Cytometry

Whole uterine horns were minced followed by collagenase (10 mg/ml) and DNase (10 mg/ml) digest. Tissues were further dispersed using an 18G needle, washed in 5% charcoal stripped fetal calf serum (CSFCS) in PBS then subsequently strained through 70 μM and 40 μM strainers. Cell suspensions were washed and incubated on ice with Mouse Seroblock FcR (Abd Serotec BUF041A; 1/100). Suspensions were then incubated with F4/80 (Abd Serotec MCA497APCT; 1/10) and/or Gr-1 (Abd Serotec MCA2387PET; 1/10) for 30 minutes on ice. To exclude dead cells, DAPI was added prior to flow cytometry analysis. Flow cytometry was performed using a BD 5L LSR Fortessa and BD FACSDiva software (BD Biosciences). Data analysis was performed using Flowjo analysis software (Flowjo LLC, Oregon USA).

### Statistical analysis

Statistical analysis was performed using Graphpad prism. Student’s t test was used to determine significance between treatments in data that were normally distributed. Non-parametric testing was utilised where sample sizes were insufficient to confirm normality of data distribution; Mann-Whitney test was used to assess differences between groups. Criterion for significance was p < 0.05. All data are presented as mean ± SEM.

## Additional Information

**How to cite this article**: Cousins, F. L. *et al*. Evidence for a dynamic role for mononuclear phagocytes during endometrial repair and remodelling. *Sci. Rep*. **6**, 36748; doi: 10.1038/srep36748 (2016).

**Publisher’s note:** Springer Nature remains neutral with regard to jurisdictional claims in published maps and institutional affiliations.

## Supplementary Material

Supplementary Information
