# Supplementary material for: Evidence for a dynamic role for mononuclear phagocytes during endometrial repair and remodelling
Source: Sci Rep. 2016 Nov 9;6:36748. doi: 10.1038/srep36748 (PMC5101509; doi:10.1038/srep36748)
Supplement: Supplementary Information [file srep36748-s1.pdf]

Evidence for a dynamic role for mononuclear phagocytes during endometrial repair and remodelling

Fiona L Cousins[‡†], Phoebe M Kirkwood[‡], Philippa TK Saunders and Douglas A Gibson*

Supplementary Data: Tables (1), Figures (5).

Species	Antigen	Supplier	Dilution
Rabbit polyclonal	GFP	Abcam ab6556	1/1000
Rat monoclonal	F4/80	eBioscience 14-4801	1/2000
Rat monoclonal	Ly6-G	Biolegend 127611	1/15000
Rabbit monoclonal	CC3	Cell Signalling Technologies #9661S	1/200
N/A	DBA lectin	Vector B-1035	1/150

Supplementary table S1. Antibodies used for immunohistochemistry.

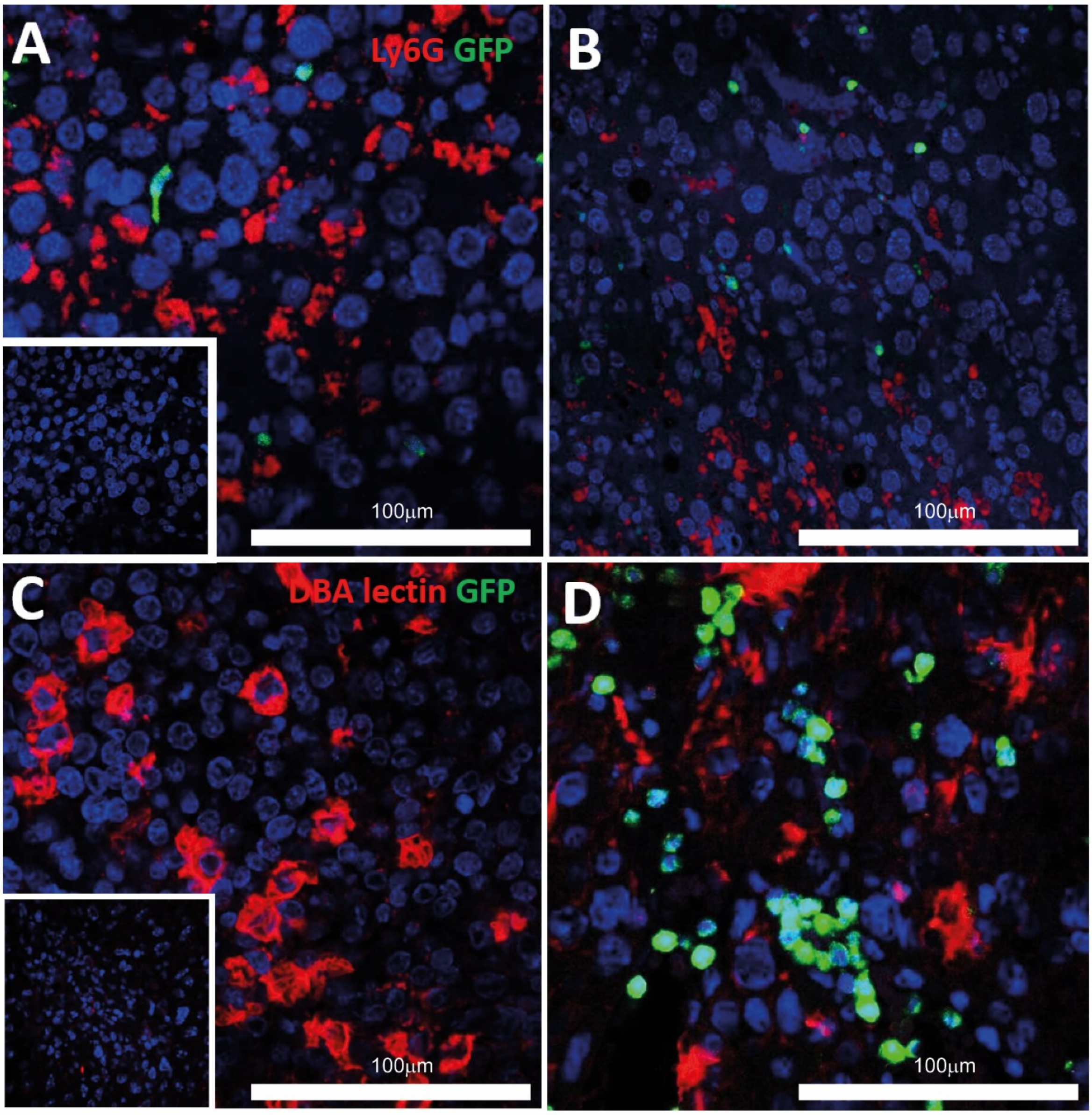

<u>Supplementary Figure 1.</u> Uterine tissues were collected at 4 hours after the withdrawal of progesterone as at this time-point uNK cells, neutrophils and macrophages were observed to be present in single stain immunohistochemistry (not shown). Putative GFP+ macrophages (green) were tested for co-localisation with neutrophil marker Ly6G (**A** and **B;** red) and uNK cell marker DBA lectin (**C** and **D;** red). No co-localisation was detected. Scale bars 100µm. Insets negative control. Images representative of three animals.

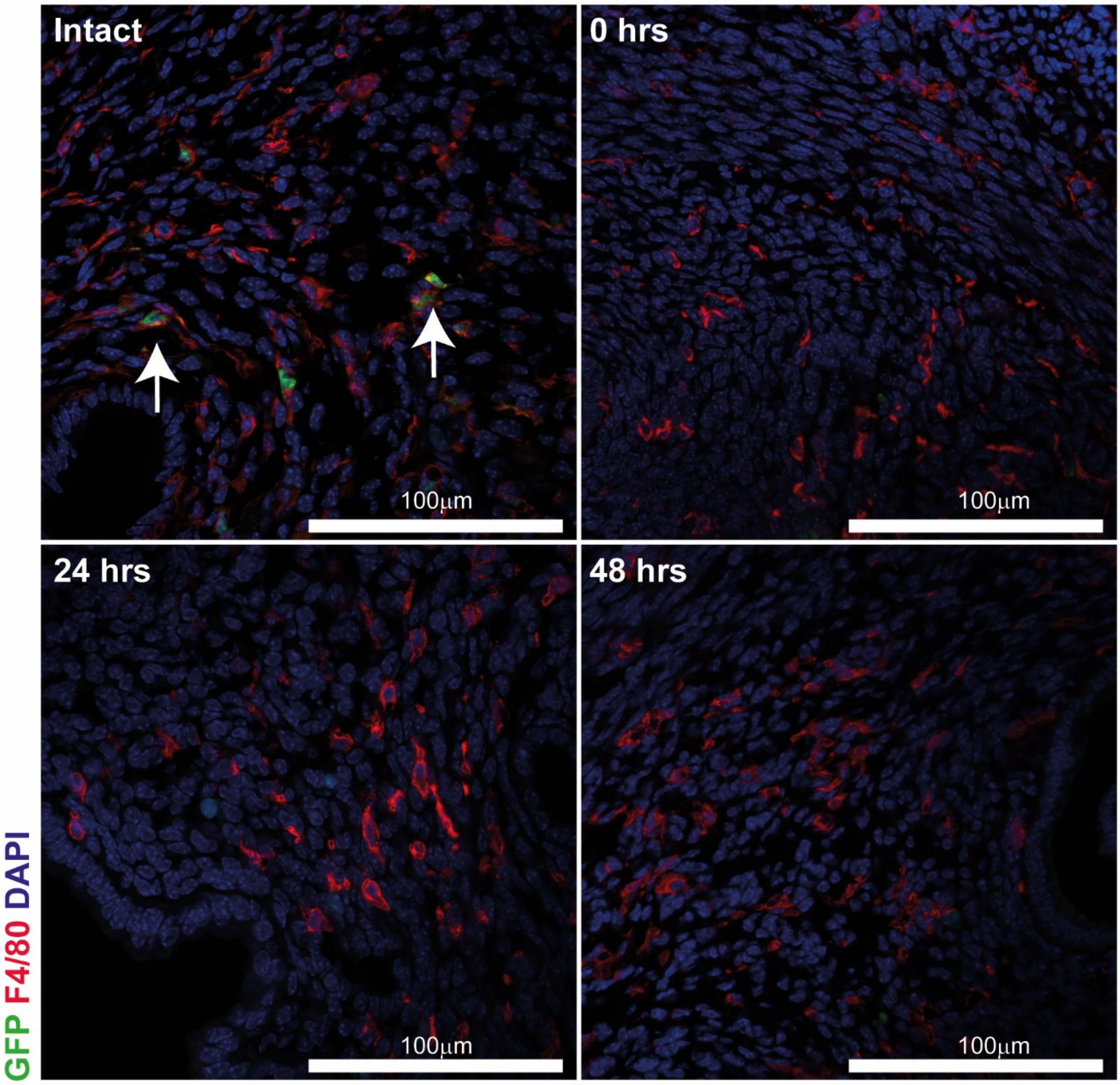

Supplementary Figure 2. Immunostaining detected abundant macrophage populations within the endometrium of both intact and unstimulated mice. The expression of GFP and F4/80 was assessed by immunohistochemistry in uteri from intact cycling mice (intact) or in the unstimulated contralateral control horn from mice across a time course of endometrial repair (0 hrs, 24 hrs, 48 hrs). Uterine tissue from unstimulated horns was exposed to the same hormonal regimen as stimulated horns but did not receive intrauterine oil stimulus. The majority of cells were immunopositive for the mouse macrophage marker F4/80 (red) and few GFP^{+} (green; arrows) macrophages were detected in the tissue at all time points. Nuclear counterstain DAPI (blue), scale bars 100µm. Images representative of three animals.

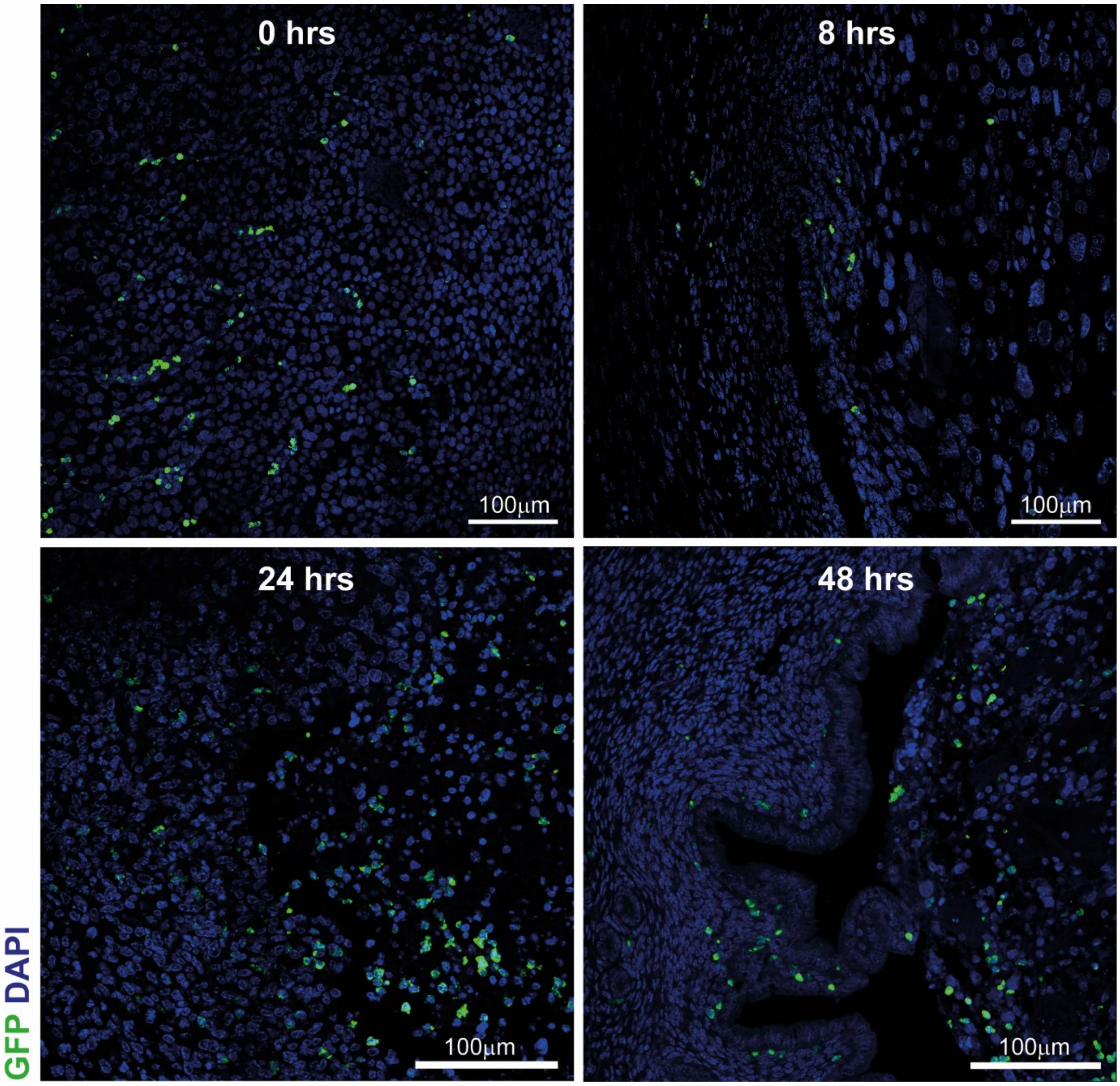

Supplementary Figure 3. The expression of GFP across a time-course of endometrial repair. The population of GFP^+ (green) cells during menstruation was assessed by immunohistochemistry in uterine tissue recovered prior to, and 8-, 24- and 48-hours after withdrawal of progesterone. Prior to endometrial breakdown (time of progesterone withdrawal; 0 hrs), in a fully decidualized mouse uterine horn, macrophages (GFP; green) were detected diffusely scattered throughout the tissue. GFP^+ cells are also detected 8 hours after withdrawal of progesterone but numbers appear unchanged. During endometrial breakdown and repair (24 hrs after withdrawal of progesterone), a dramatic influx of GFP^+ cells is apparent throughout the uterus. At 48 hrs endometrial repair is complete and re-epithelisation of the luminal surface was apparent. GFP^+ cells persist and are detected close to the restored epithelial surface. Nuclear counterstain DAPI (blue), scale bars 100µm. Images representative of three animals.

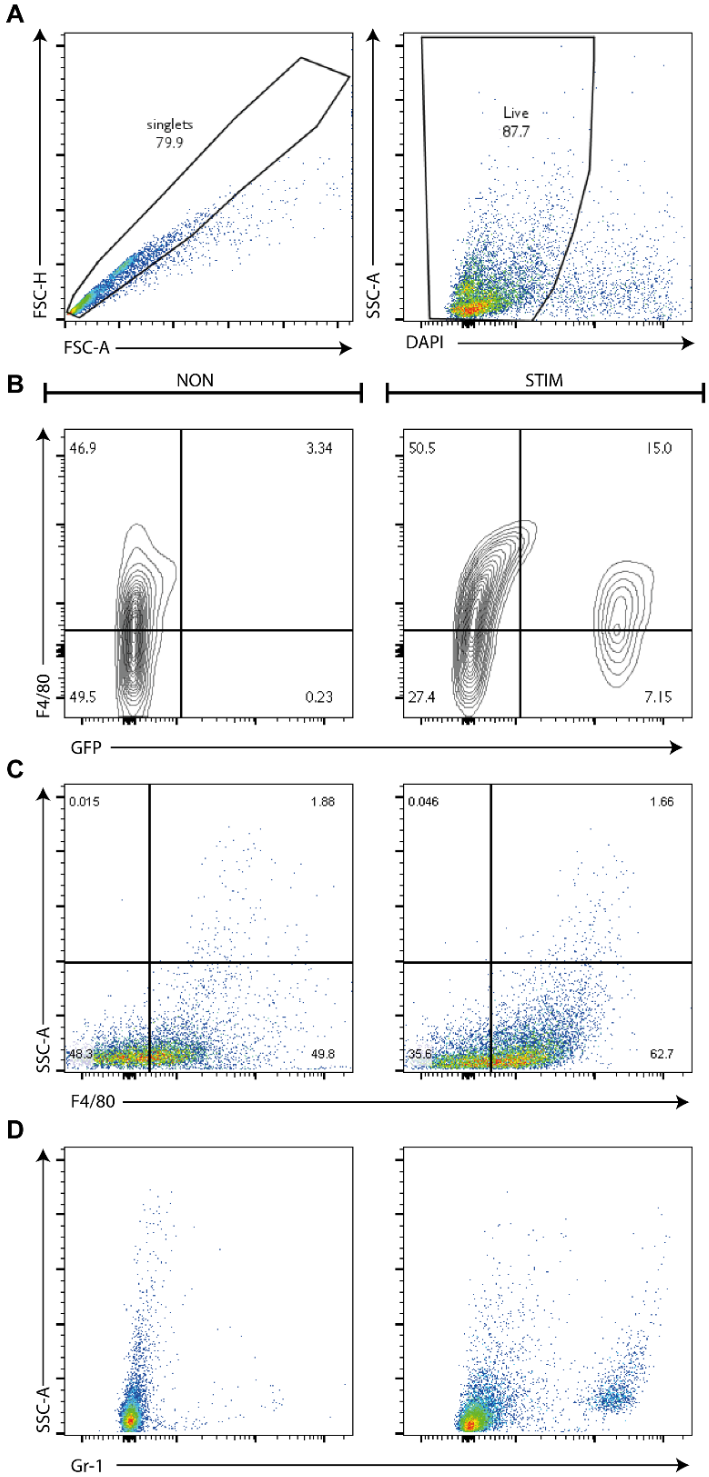

<u>**Supplementary Figure 4.**</u> Immunophenotyping of uterine macrophages 24 hours after withdrawal of progesterone. **A** All uterine cell samples were live singlet gated; live cells were identified by DAPI exclusion. Flow parameters of cells from unstimulated (non) and stimulated (stim) uterine horns were compared (B-D). **B** In MacGreen mice, few GFP^+ cells were detected in non-stimulated uterine horns which was in marked contrast to the abundant GFP^+ cells in the decidualized horn. **C** Murine eosinophils are also reported to express F4/80 but do not require CSF-1 for differentiation. Flow cytometry analysis demonstrated that the majority of $F4/80^+$ cells had low granularity (SSC) precluding eosinophils and confirming macrophage phenotype. **D** In wild type mice, a comparable influx of $Gr\text{-}1^+$ cells was detected in decidualized horns.

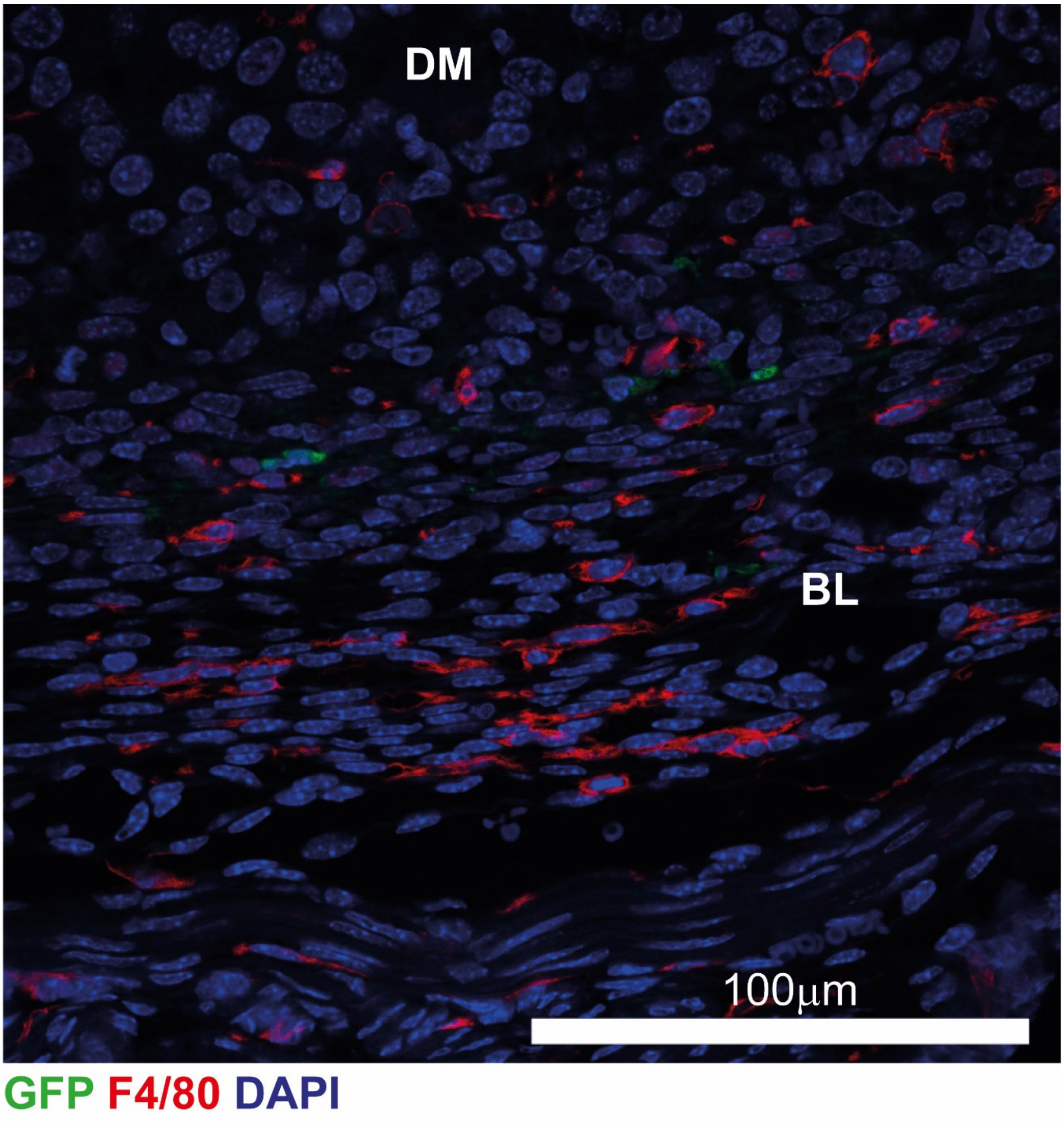

Supplementary Figure 5. Immunostaining detected abundant macrophage populations within the endometrium of stimulated mice at the 0 hour time point. The expression of GFP and F4/80 was assessed by immunohistochemistry. The majority of cells were immunopositive for the mouse macrophage marker F4/80 (red) but few GFP^+ (green) cells were detected in the tissue at this time point. F4/80 positive cells were abundant in basal layer (BL) and were also detected within the decidualised mass (DM). Nuclear counterstain DAPI (blue), scale bars 100µm. Images representative of three animals.
